# Double Deep Q-Learning and Faster R-CNN-Based Autonomous Vehicle Navigation and Obstacle Avoidance in Dynamic Environment

**DOI:** 10.3390/s21041468

**Published:** 2021-02-20

**Authors:** Razin Bin Issa, Modhumonty Das, Md. Saferi Rahman, Monika Barua, Md. Khalilur Rhaman, Kazi Shah Nawaz Ripon, Md. Golam Rabiul Alam

**Affiliations:** 1 Department of Computer Science and Engineering, School of Data and Sciences, BRAC University, 66 Mohakhali, Dhaka 1212, Bangladesh; razin.bin.issa@g.bracu.ac.bd (R.B.I.); modhumonty.das@g.bracu.ac.bd (M.D.); md.saferi.rahman@g.bracu.ac.bd (M.S.R.); monika.barua@g.bracu.ac.bd (M.B.); khalilur@bracu.ac.bd (M.K.R.); rabiul.alam@bracu.ac.bd (M.G.R.A.); 2 Faculty of Computer Sciences, Østfold University College, 1783 Halden, Norway

**Keywords:** autonomous vehicle, reinforcement learning, Double Deep Q Learning, faster R-CNN, object classifier, markov decision process

## Abstract

Autonomous vehicle navigation in an unknown dynamic environment is crucial for both supervised- and Reinforcement Learning-based autonomous maneuvering. The cooperative fusion of these two learning approaches has the potential to be an effective mechanism to tackle indefinite environmental dynamics. Most of the state-of-the-art autonomous vehicle navigation systems are trained on a specific mapped model with familiar environmental dynamics. However, this research focuses on the cooperative fusion of supervised and Reinforcement Learning technologies for autonomous navigation of land vehicles in a dynamic and unknown environment. The Faster R-CNN, a supervised learning approach, identifies the ambient environmental obstacles for untroubled maneuver of the autonomous vehicle. Whereas, the training policies of Double Deep Q-Learning, a Reinforcement Learning approach, enable the autonomous agent to learn effective navigation decisions form the dynamic environment. The proposed model is primarily tested in a gaming environment similar to the real-world. It exhibits the overall efficiency and effectiveness in the maneuver of autonomous land vehicles.

## 1. Introduction

Autonomous vehicles, in this modern era, are a vital part of an advanced transportation system. Autonomous vehicles are considered to be one of the fastest-growing technologies that exist at present. The autonomous vehicle extracts environment perception to conclude directing the agent [1]. Decision-making is the main module of an autonomous vehicle. Thus, it is vital to make an autonomous vehicle learn finding an optimal path for traversing. This work suggests the integration of Reinforcement Learning method in an autonomous vehicle to make it able to take optimal decisions while traversing in a dynamic environment.

Reinforcement Learning is a kind of machine learning algorithm that works with gaining experiences through communicating with the worldly environment and evaluating feedback to develop a system’s performance to make behavioral decisions [2]. It improves the system’s performance through trial and error experience with a dynamic environment. Reinforcement Learning provides qualitative and quantitative frameworks through rewards and punishment to understand and adapt decision-making [3]. The decision-making executes a particular operation through maximizing reward in a specific circumstance [4]. Incorporated with several machines and software, it looks for the best possible behavior that it should take in a particular condition. Usually, the agent in a Reinforcement Learning model communicates with the environment through perception and action [5]. It inputs indication from the environment, and the agent takes actions based on decisions which are generated as output. Thus, Reinforcement Learning involves learning to take decisions, mapping situations to actions and maximizing reward signals [6].

A Reinforcement Learning-based agent decides how a given task will be performed on its own from a training data-set. However, in the absence of a training data-set, an agent has to learn from its experience. For learning to take optimal decisions, the vehicle must explore the same environment many times. A balance of exploitation and exploration is thus needed to get the agent to learn finding better goals [7]. Exploitation is what the agent already knows about the worldly environment and what it knows of as the best results [7]. On the other hand, exploration is to discover new conditions and features of the world and finding a better goal path than what the agent knows of already. The typical autonomous vehicle systems are limited within specific mapped models. Including Reinforcement Learning model in autonomous vehicles will make the agent able to operate in a dynamic environment through exploitation and exploration. In consequence, autonomous vehicles will be able to make path finding decisions to traverse in even unknown environments [8].

In this work, we use Double Deep Q-Learning [6] as the Reinforcement Learning algorithm for the agent to explore the environment. While the Q-Learning algorithm thrives on finding adequate measures to decide in a provided condition, the Double Q-Learning solves the problem of overestimation of Q-value in basic Q-Learning [9]. The precision of Q-values depends on the actions that are attempted and the states that are explored. Hence, an agent does not have necessary information regarding which action to take at the beginning of the training. Choosing the maximum Q-value as the best action can give false positive results and can be noisy. Thus, Double Deep Q-Learning Network uses two separate networks which are Deep Q Network and Target Network to dissociate selection of action from target Q-value generation. As a consequence, DDQN significantly helps in reduction of overestimation of Q-values which assists an agent in steady learning through faster training and proves this method to be better than other learning algorithms. Therefore, DDQN proves to be suitable for an autonomous agent to make decisions for optimal traversing by picking the maximum Q-value while exploring the same environment. The process in which the agent consistently determines the maximum Q-value for navigation is called the epsilon greedy strategy [6].

This work also includes detecting and classifying obstacles along its way while navigating. It takes data from obstacles in rocky, rough and bumpy surfaces through the sensor. We have mainly used a vision sensor to implement the mentioned proposal. The sensory data are fed to the agent, and the decision is taken based on the fed conditions. Faster R-CNN [10] is primarily applied for the prototype as the vehicle tries to identify and detect objects while navigating. Faster R-CNN, currently, is a distinguished algorithm for object classification. The R-CNN and Fast R-CNN algorithms follow selective search algorithm to detect the region proposals. However, in Faster R-CNN [11], selective search algorithm is eliminated and the network itself learns the region proposals making this algorithm better than its predecessors. It utilizes convolutional network for region proposal and object detection, making it swifter and suitable in case of real-time object detection.

**Figure 1:**
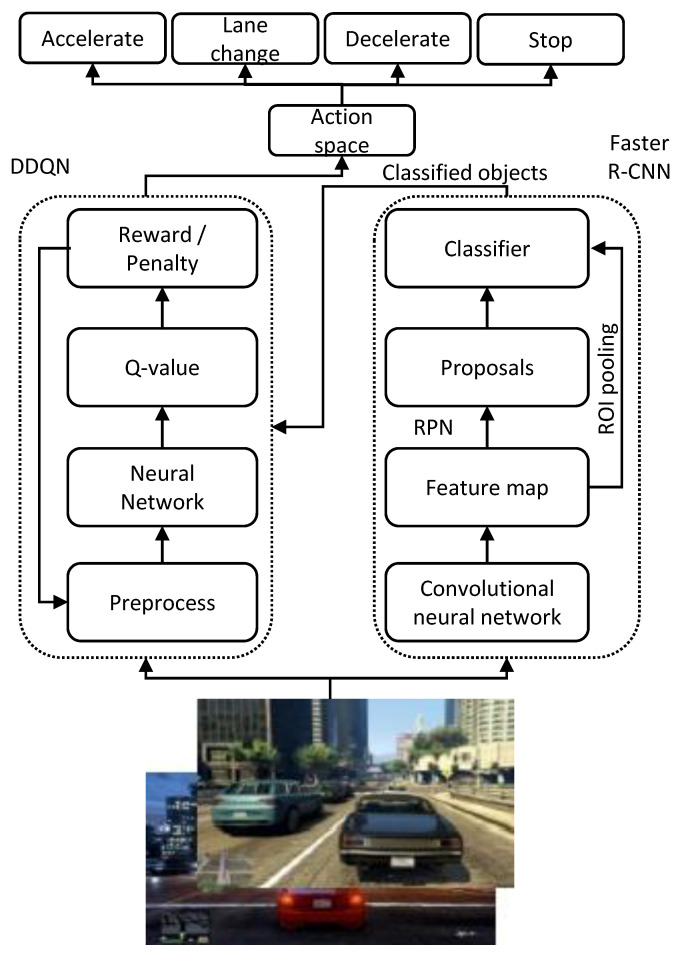
Proposed architecture of autonomous vehicle navigation and obstacle avoidance in dynamic environment.

Several highway decision-making strategies [12,13] have been performed with deep Reinforcement Learning; in this research, the deep Q-Learning approach is incorporated with the Faster R-CNN method so that an autonomous agent can also detect and avoid obstacles along its way while traversing. Although the deep Q-Learning and Faster R-CNN algorithms have proven to be successful for autonomous driving strategy and object classification, respectively, the fusion of these two methods for autonomous maneuver combines the benefits of these two approaches in autonomous vehicle navigation. Figure 1 shows the proposed autonomous vehicle model based on Reinforcement Learning. The proposed model merges the Double Deep Q-Learning Network (DDQN) and Faster R-CNN, and integrates into an autonomous vehicle so that it can make maneuvering decisions while classifying and avoiding objects and obstacles on its way. The proposed model is tested on a gaming environment that is similar to the real-world scenario.

The major contributions of this research work are as follows:

- This research presents the development of two learning approaches algorithms, i.e., a combination of Double Deep Q-Learning and Faster R-CNN for an autonomous vehicle in order to identify obstacles and navigate properly. Therefore, it integrates the benefits of these two approaches and ensures autonomous navigation and obstacle avoidance in a stochastic vehicular environment.
- Real-world testing of algorithms on autonomous vehicles is time-consuming and expensive; therefore, a dynamic game engine simulator is used for training and validating the proposed model.

This paper is organized as follows. Section 2 presents the related works. Section 3 discusses the object classifier methodology through Faster R-CNN. The application of Reinforcement Learning in the autonomous vehicle is described in Section 4. In Section 5, the implementation is described in detail. Section 6 contains the experimental results with analysis. Section 7 presents the discussion. Finally, Section 8 concludes the paper.

## 2. Related Works

A primary proposition of object detection is classifying few interesting regions and use the Convolutional Neural Network (CNN) to it [14]. Erhan et al. propose the Region-based Convolutional Neural Networks (R-CNN) to minimize the interface by focusing on a single region at a time [10]. In this work, we use the Faster R-CNN to make object detection more efficient. Other image classifying algorithms, such as R-CNN, apply the selective search for regions; however, Faster R-CNN applies a separate network to predict region proposals. The bounding boxes value is projected by reshaping the proposed region. In [4], the authors used a region-based CNN using deep learning for road obstacles detection [15]. However, our proposed model does not only let an agent identify obstacles; it also determines actions based on them by integrating DDQN.

Min et al. present a related work which influences driving policy on highways using Reinforcement Learning [16]. Their proposed model involves training a Driving Assistance System Supervisor by deep Reinforcement Learning. In our proposed model, we have used a game engine simulator that differs from the driving simulator used in [16]. The driving simulator used in [16] is implemented by Unity ML agents for static environment while we have implemented our system in a GTA V dynamic game environment. Real-world testing of algorithms on autonomous vehicles is time-consuming and expensive, so the authors of [17] present a visually and physically realistic simulator and tested it on a quadrotor autonomous agent. However, as mentioned earlier, we used a game engine as a simulator to test our algorithm. This is because the scenario of that game is almost similar to the real-world. Identical to our proposed approach, Reinforcement Learning has been used as a decision-making method in [18]. It focuses on combining longitudinal and lateral control in traffic overtaking maneuver. In our work, the maneuvering decisions include lane changing along with acceleration, deceleration and stopping as needed in a dynamic environment.

As human-driven vehicles and autonomous vehicles coexist on land, efficient maneuver of autonomous vehicles has become a necessity. In [19], a regret theory is adapted based on human drivers’ lane-changing behavior. The predicted decision is integrated, and DDQN is used in training the autonomous vehicle controller. Our proposed model varies as we train the autonomous vehicle to determine path based on the distance calculated in real-time from other vehicles or obstacles. In [20], the author presents longitudinal control of autonomous land vehicle models by using parameterized Reinforcement Learning. It is mostly implemented using PBAC algorithm and is different from the Double Deep Q-Learning algorithm that we have used in our previous work [21]. The authors in [22] depict eight extensions of Reinforcement Learning which consist of adaptive heuristic critic (AHC) learning, Q-Learning and three further extensions to the basic methods to accelerate learning. Our proposed model focuses on Deep Reinforcement-based Learning to train the autonomous agent throughout. In [23], the authors present a Reinforcement Learning-based approach for autonomous helicopter flight where a dynamic model is created first by the help of a pilot flying the helicopter. The approach, later, integrates with Differential Dynamic Programming (DDP) to learn a controller for optimization of the model. Our application of Reinforcement Learning to land vehicles works in a way that the proposed model takes data from a gaming environment while traversing and learns to take decisions with Double Deep Q-Learning algorithm.

Thus, Reinforcement Learning is considered as an exciting learning method which requires performance feedback from the environment. So far, Reinforcement Learning solved various learning problems. This paper explores Reinforcement Learning as a decision-maker for maneuvering and path-finding in any environment. The proposed work proves to be promising as it not only implements a decision-making method based on Double Deep Q-Learning for autonomous vehicles but also it integrates Faster R-CNN. Ultimately, our proposed model can avoid obstacles while traversing in a dynamic environment. The Double Deep Q-Learning algorithm is used to train the autonomous vehicles for navigation control. The autonomous agent makes path-finding decisions on its own by avoiding obstacles as the distance from obstacles is calculated real-time to take maneuvering decisions which include lane-changing, accelerating, decelerating and stopping.

## 3. Object Classification Using Faster R-CNN

### 3.1. Faster R-CNN

The R-CNN operates in a distinctive way to trim the computed box proposition of a remotely featured input picture and implements neural classifier on it. Unlike other techniques, it costs much as it requires many crops which creates overlap calculation. However, the use of Fast R-CNN can curtail this problem. Fast R-CNN sends the whole picture to feature extractor and trims the picture from a middle layer and smooths the highlighting extraction process. Prior, R-CNN and Fast R-CNN required outer proposition generator. Now, the neural network can do the same work more efficiently. On the picture, it is normal to have boxes on the beat of each other which are mainly called ‘’anchors’’. Scales and perspective proportions are required to maintain an outline. Thus, to anticipate each anchor, a model is designed by (*i*) expectation for a discrete class of individual anchor, and (ii) an accumulation of counterbalance forecast which will be required to relocate the anchor for fitting in the ground truth bounding box. A consolidated distribution and relapse loss are discussed below.

As part of the process for each anchor *a*, there is a best matching ground-truth box *b*. If the identification process is completed and the best match is found, then it will be called a “positive anchor”. It is accredited with (*i*) class specify $y_{a}\epsilon${1 ...k}, and (ii) the best coordinating empirical evidence box *b* is first to be established for individual anchor *a* (called the crate concealing Φ($b_{a}$; *a*)). If no such match could be discovered, it has been considered as a “negative anchor”, and set the class name to be $y_{a}=0$. Thinking about the anchor as *a*, on the off chance that we foresee box concealing $f_{loc}$ (*I*; *a*; θ) and relating class $f_{cls}$ (*I*; *a*; θ), where θ represents the model boundaries and I is the picture, at that point the loss for I is estimated as a weighted amount of a classification loss and an area-based loss:

(1) $$\begin{array}{c} L\left(a;I;\theta \right)=\alpha \times 1\left[aispositive\right]\times l_{loc}\left(\Phi \left(b_{a};a\right)-f_{loc}\left(I;a;\theta \right)\right)+\beta \times l_{cls}\left(y_{a},f_{cls}\left(I;a;\theta \right)\right) \end{array}$$

Here α, β refers to weights which balance classification losses and localization, respectively. Equation (1) is reduced with respect to parameters θ [24] and to train the system, it is averaged over anchors.

Identifying anchors has excellent repercussions for its accuracy and computation. Unlike calculating the anchors from the data-set using clustered ground-truth boxes, now it is conducted by tiling boxes over the image with respect to various scales and ratios. The vital side of using a network is that the tiled indicators of the picture, along with its shared parameters, can be used to compose the forecast. It stands out as a traditional sliding window method [11,25].

#### Acquiring Data-Set

A huge data-set named *Open Image V5* [26] is used to conduct experiments. It is produced by Google, and also it is a free index. It is enriched with elucidated images which have 600 box-able object classes. There are total 1,743,042 training images which include bounding boxes, visual relationship, object segmentation, total validation (41,620 pictures) and test sets as well. However, not all 600 classes are required for our Object Classifier.

Seven distinctive classes (Motorcycle, Van, Bus, Bicycle, Car, Person, Truck) are separated from these 600 classes using ‘OIDv4_ToolKit’. To train our model, 8335 images are extracted, where each class contains at least 1000 images. For testing, a total of 2360 images are extracted from the data-set, where each class contains at least 300 images. Then, labels of these images are accumulated in *.XML* format.

### 3.2. Training Object Classifier

TensorFlow framework [27] is used to run a feature separator, which is called Faster R-CNN Inception V2 [28], to train the data-set and to prepare the object classifier as well. NVIDIA GTX 1050 GPU featured a computer, with CUDA core reinforce has been utilized for developing and testing methods. The training process was run for 18 h at a stretch until the total loss was under 0.4. Image classifier was prepared by conducting a total of 200,000 steps.

Faster R-CNN involves two phases to complete the detection method. The Region proposal Network (RPN) is the primary phase which processes images through feature extractor. In this model, the feature extractor (Faster R-CNN Inception V2) has been utilized at the transitional level to foresee class analytic box proposition. Additionally, loss function of initial stage shows up as the Loss Equation using a matrix of anchors tiled in scale, space and angle proportion.

**Figure 2:**
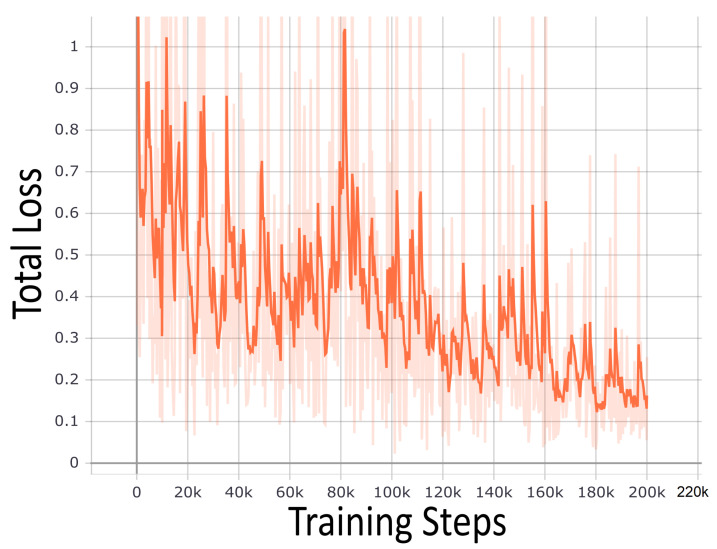
Absolute loss diagram of the developed data model.

In the following stage, utilizing the forecast of box proposition is used to manage highlights from the element map. At that point, these highlights are utilized in the remainder of feature extractor for every proposition to anticipate a class and for refining class-explicit box. The loss function during the current second stage box classifier in like manner shows up as the Loss Equation utilizing the proposition created as anchors from the RPN. Figure 2 demonstrates the standardized type of absolute loss diagram.

## 4. Distributional Agent for Autonomous Driving

### 4.1. Double Deep Q-Learning Network (DDQN)

H. V. Hasselt came up with the idea of DDQN [9] as an extension of his past proposition which applies to Deep Q Network (DQN) [29]. The DQN is one of only a handful few Q-Learning-based methods. Estimation errors cause overestimation issues among these Q-Learning-based algorithms. Overoptimistic fee evaluation and performance decrepitude occur because of overestimation. In any case, the system for DDQN does not merely lessen the overoptimistic revere evaluation, yet also gives preferred execution over DQN on a couple of virtual accustoms. Excerpt and interpretation measures are disengaged by DDQN while it focuses on two Q-functions as a motivation. The objective regard states of DDQN and DQN are:

(2) $$y^{DQN}=R_{t}+\gamma \max_{A_{t+1}}Q\left(S_{t+1},A_{t+1};\theta^{-}\right)$$

(3) $$y^{DDQN}=R_{t}+\gamma Q\left(S_{t+1},\underset{A_{t+1}}{\mathrm{argmax}}Q\left(S_{t+1},A_{t+1};\theta \right);\theta^{-}\right)$$

### 4.2. Markov Decision Process for Path Circulation

Markov Decision Process expresses the securing way course for self-governing driving in this analysis. The actor determines his activity in each progression, and quickly a prize is earned for that response. The tuple {S, P, A, R, γ} which has just been articulated previously chronicles Markov Decision Process (MDP). For better understanding, a short synopsis of MDP is expressed below:

- s ϵ S defines the limited state area which accommodates a gray proportioned picture from vision sensors of the actor.
- $P\left(s^{\prime }∥s\;a\;)\;:S\times A\times S\to \left[0,1\right]\right.$, where P defines the evolution behavior.
- a ϵ A is definite response area which works for an actor.
- R defines reward behavior, where R(s,a):S×A→R
- γ characterizes the rebate aspect, where γ→ [0,1] for deferred reward.

**Figure 3:**
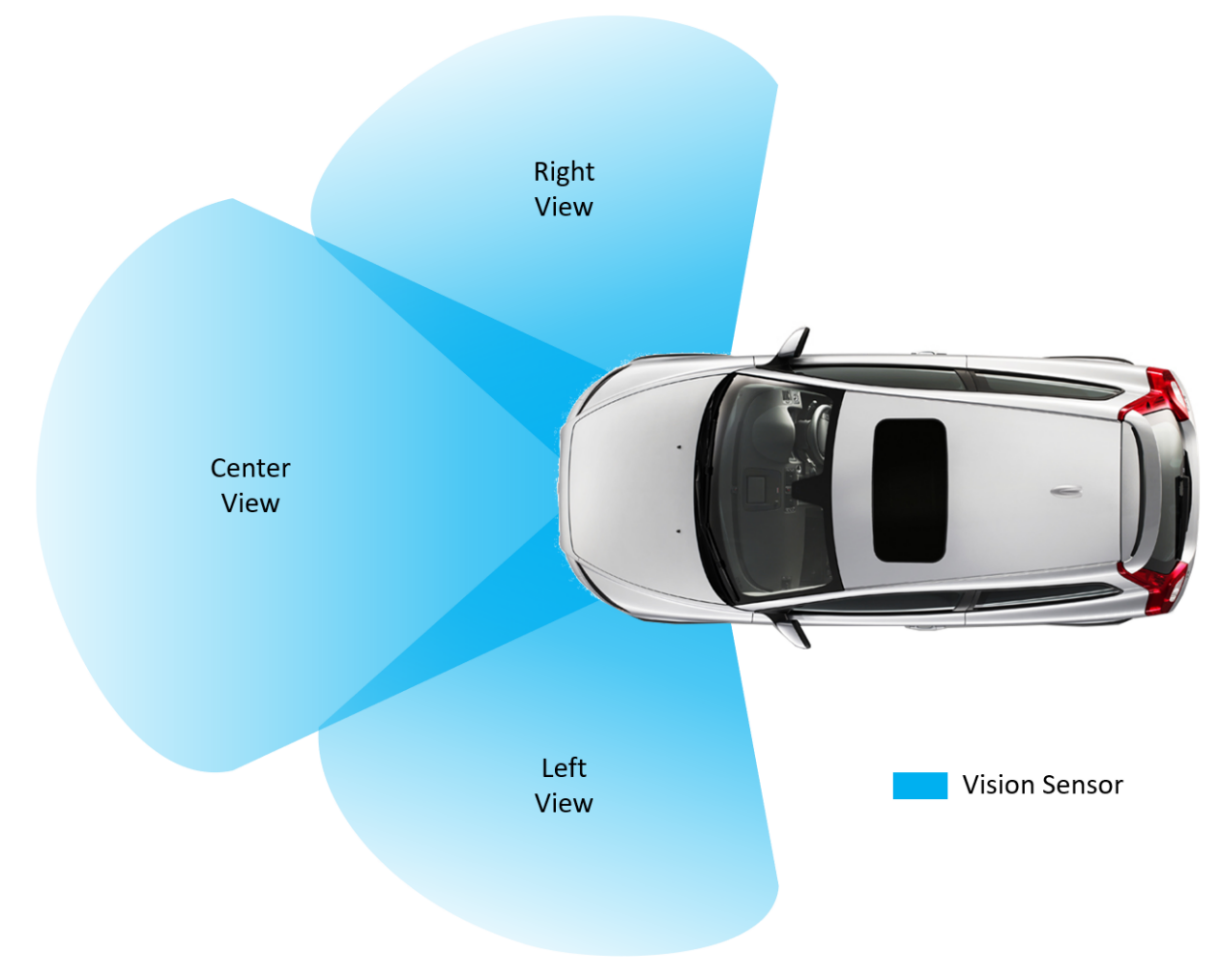
Vision sensor coverage of perception

For the high dimensional perceptions, MDP states *s*ϵ*S* can be utilized by adopting Deep Neural Networks [30]. Figure 3 speaks to the view of the encompassing inclusion by adopting three vision sensors placed in the front.

The autonomous driving actor has five particular activities. The definite response area *A* comprises of forward, left, right, stop and deceleration. For forward and deceleration, 5 kph is summed or deducted from the running actor acceleration. The actor acceleration is bound in the scope of 30 kph to 80 kph. Actor naturally changes the acceleration for vehicles in a specific separation so that it keeps up a protected gap from the front vehicle. When the vehicle in front out of nowhere slows down or some other vehicle cuts in suddenly before our representative vehicle, the ’stop’ action appears immediately.

### 4.3. Data Preprocessing

The images of surroundings are collected from three vision sensors, as shown in Figure 3. The output pictures from vision sensors are edited so the model will not be prepared with the sky and the vehicle’s forward portions. According to NVIDIA model, those pictures are converted to 160 × 320 (3 YUV channels). Those pictures are standardized (picture information isolated by 127.5 and deducted 1.0). As expressed in the Model Scheme area, this is to maintain congestion and make gradients work enhanced.

### 4.4. Model Architecture Pattern

Planning the discernment state, *S* and taking the accompanying action on response area, *A* is the primary objective of the actor, π(a]s). The total of the action will be driven in a stochastic driving situation. Be that as it may, to achieve this planning the model necessities to fulfill to specific conditions: (*i*) concentrate and catch huge features from three vision sensor’s pictures, and (ii) it should assess the characteristic arbitrariness of the climate for picking a specific activity.

The network should detect spatio-balanced data recovering from vision sensors to fulfill the primary condition. Utilizing CNN directs this cycle. CNN is well-known for extricating spatial aspects in distinction to pictures. Additionally, immense spatial vision sensor pictures are polished into ocular component vector utilizing two-dimensional three convolutional layers.

In addition, the subsequent situation can be satisfied by utilizing the DDQN scheme. Stochastic driving conditions utilize this scheme. For every activity, there is a restoration circulation made by the totaFull connected layer with the assistance of θ. The *Q*(*s*,*a*) decision can be assessed as the longing of quantiles, $\sum_{i}q_{i}\theta_{i}\left(s,a\right)$.

Furthermore, the most extreme Q-value perhaps recovered from the highest response, $a^{*}$ that can be additionally selected from open restricted Q-values of response area, *A*.

(4) $$a^{*}={\mathrm{argmax}}_{a}Q\left(s,{\mathrm{argmax}}_{a}Q\left(s,a\right)\right)$$

**Figure 4:**
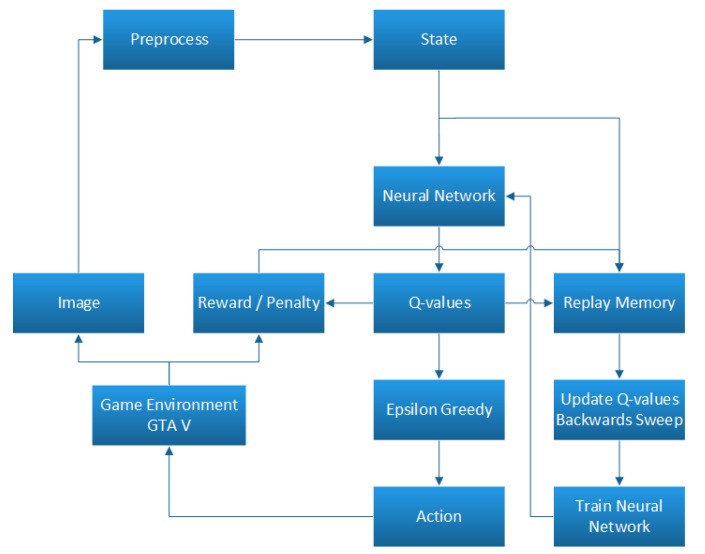
Flow diagram of recommended DDQN scheme.

The flow diagram of recommended DDQN scheme for the proposed algorithm is presented in Figure 4. We use Keras to prepare this proposed network.

### 4.5. Hyperparameters

The network is constructed after NVIDIA model. It is used to execute start to finish an autonomous test by NVIDIA. The NVIDIA model itself is all around archived. Supervised image distribution or relapse issues can be fathomed inconsistent strategy utilizing the deep convolutional network. Accordingly, the primary spotlight lies on changing the preparation pictures for conveying the best outcome. In any case, to procure the best outcome, fundamental changes have been prepared for abstaining from over-fitting nature and including impartiality for preparing the forecast precise. Moreover, the accompanying acclimation has been summed to the model.

- Lambda layer is acquainted with standardizing input pictures for preparing gradients to work all the more easily and to dodge saturation.
- Extra dropout layer is included following the convolution layers for staying away from the over-fitting situation.
- At that point ReLU has been executed for actuation capacity to guarantee linearity.

Adam Optimizer at a 1 × 10${}^{-5}$ learning rate with epsilon 0.0001 and 32 set of mini-batches is utilized for preparing the network to get superior precision. For instating network loads, Xavier Initializer has been utilized, and all data sources are standardized into [1,−1]. To accomplish the precision of the forecast of guiding plot for every picture, mean squared errors have been utilized to assess the loss function. Table 1 shows the hyper-parameters of this propulsive approach network. Estimation of support Q as 200 has been set. Replay memory’s value is 5,000,000, and γ is used as the markdown aspect, which has been locked to 0.99. ϵ-greedy policy has been utilized where ϵ was progressively reduced to 0.1 from 1.0 in every progression and afterwards locked to 0.1. Those strategies have been actualized during 3 millions of stages training.

### 4.6. Model Training

Agent training has been done on the game environment and based on the pictures collected from the cameras of the agent. The study assumptions includes the daylight for clear vision and known objects for classification. However, for diversification, we utilized the accompanying growth procedure alongside the Python generator to produce a limitless number of pictures. These pictures have been used arbitrarily as well as changing the characteristics of those images for giving the agent various kinds of scenarios like changing images splendor and shadows, flip images between left and right, etc. Arbitrary detail changes have been given below:

- Arbitrarily select right, center or left picture.
- Steering angle is adapted by +0.2 for left picture.
- Steering angle is adapted by −0.2 for right picture.
- Arbitrarily flip picture right/left.
- Arbitrarily convert picture horizontally with steering angle accommodated (0.002 per pixel shift).
- Arbitrarily convert picture vertically.
- Arbitrarily added shadows.
- Arbitrarily changing picture splendor (lighter or more obscure).

Utilizing the left/right pictures is valuable for preparing the recuperation driving situation. The level interpretation is helpful for troublesome bend taking care of.

## 5. Implementation

This section explains the implementation procedure of the proposed model for navigation of autonomous vehicle and obstacle avoidance in a dynamic environment. We implement and test the object detection part of the model using the Open Image V5 data-set [26]. On the other hand, the reward determination part of the model is implemented and tested on GTA V game environment. The implementation of the models consists of three stages: (*i*) object detection using Faster R-CNN, (ii) reward determination using Reinforcement Learning algorithm and (iii) Double Deep Q-Learning and combined decision-making.

### 5.1. Object Detection

We take the translated image from the three physical vision sensors of the autonomous vehicle and run the image through the image classifying algorithm architecture. It uses the Fast R-CNN as the detector, which includes CNN backbone, RoI pooling layer and fully connected layers for bounding box regression and classification. Firstly, a feature map for the image is generated through the backbone CNN. The Region Proposal Networks have bounding box proposals that are then utilized for pooling features from the feature map. RoI pooling is very advantageous to be used here. The features are provided to the sibling classification and regression branches. Passing through a softmax layer, they obtain classification scores. These scores determine the belonging class of each box. Predicted bounding boxes are developed utilizing the regression layer coefficients.

### 5.2. Reward Determination

**Figure 5:**
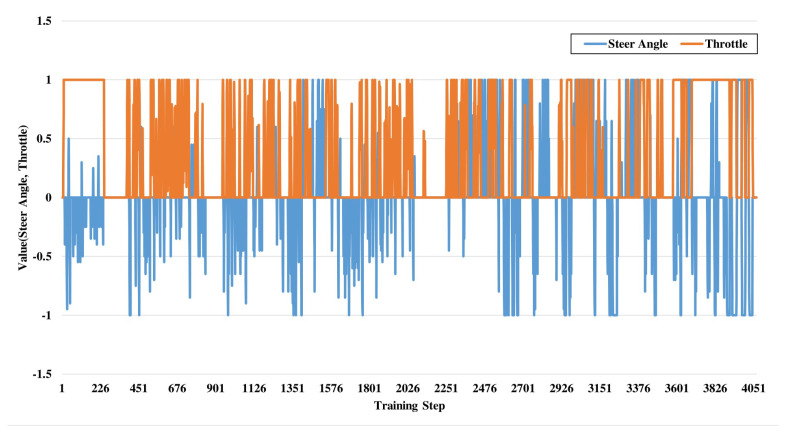
Training data model object classification: steering and throttle angle.

**Figure 6:**
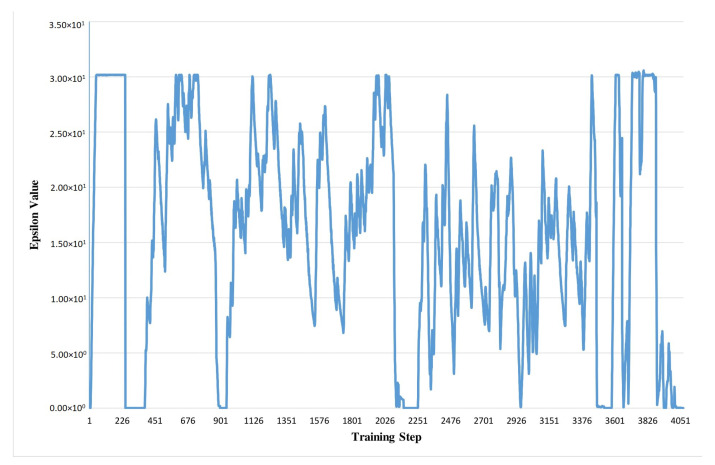
Training data model object classification: epsilon value.

Double Deep Q-Learning works based on reward value which is evaluated from the reward function of this algorithm. The main part of this reward function is epsilon value determination. These epsilon values are generated in the data pre-processing part. In the training period, we generate a CSV file from input data. Figure 5 shows the steering angle and throttle value during the model object classification portion. This data-set file consists of 3 image files of 3 vision sensors which have been already discussed in Section 3 and Section 4. The steering angle is calculated from each image during the training period as well as throttle value which is in −1 to +1 range. After that, the training procedure also generates the epsilon value for each case which helps to construct the reward values as shown in Figure 6. Steering angle values and throttle values are the vital actuators to control autonomous vehicle. If the RL agent observes no obstacle in front of it then it increases its throttle value gradually. On the other hand, if it detects any obstacle then it reduces its throttle and changes the steering angle so that it can avoid the obstacle. The relation of steering angle and throttle value has been shown for a testing period. Reward values in Reinforcement Learning refer to the numeric value that an agent receives for performing right actions to the environment using actuators. An agent’s aim is to maximize its entire reward in order to learn certain behavior. The evaluated reward values are fed to the agent after each training, and they determine the action taken by an autonomous agent. The reward policy makes the process of decision-making more precise.

### 5.3. Combined Decision Making

**Figure 7:**
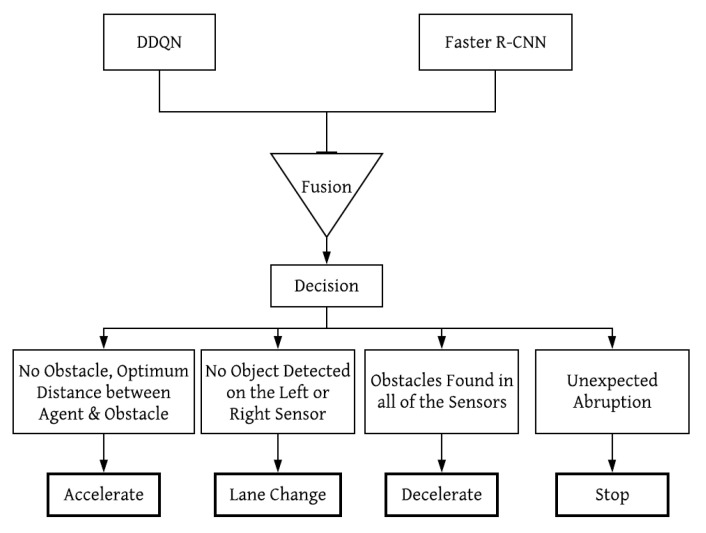
Combined decision-making process.

The flow diagram in Figure 7 represents the decision-making procedure that the agent follows. The agent takes the decision based on the output result from the DDQN and Faster R-CNN fusion. DDQN assists an autonomous agent to generate and choose actions to achieve the maximum rewards. Here, Faster R-CNN outputs object classification from input images from the environments which influences the reward values generated from DDQN and helps to get more accurate results even in case of randomness. The DDQN agent can come to a decision regarding what action should be appropriate for a particular situation based on the explored environment and Faster R-CNN outputs. Here, four decisions or actions are considered for different scenarios. Therefore, an agent’s response and decision-making are categorized into accelerate, lane change, decelerate and stop modes. Generally, if the agent finds no obstacle in a certain distance, it will be in acceleration mode. If no obstacle is found on the right or left side of the agent, then it will be able to change its lane. It will be in deceleration mode if obstacles are found on both sides of the agent. Lastly, it will immediately stop its exploration in case of any unexpected abruption.

An agent takes decisions based on the distance calculations of surroundings and the condition which satisfies the action. Moreover, to make the decision-making process error-free and smooth, an agent calculates the braking distance precisely. The equation for calculating the braking distance is as follows:

(5) $$\;\mathrm{Distance}\;=\frac{1}{2}\times \frac{V_{\mathrm{init}\;}^{2}}{\mathrm{Gravity}}$$

## 6. Results

The integration of Faster R-CNN and DDQN shows the optimum result in autonomous exploration. The data-set that is used here to train the image classifier shows the accuracy of 94.06%. This accuracy level represents the effectiveness of the classifier to identify any object which may appear before the autonomous vehicle. Acquiring values from Faster R-CNN, the reward function can be manipulated, which accelerates the efficiency in the decision-making process and enables safe autonomous exploration of vehicle.

**Figure 8:**
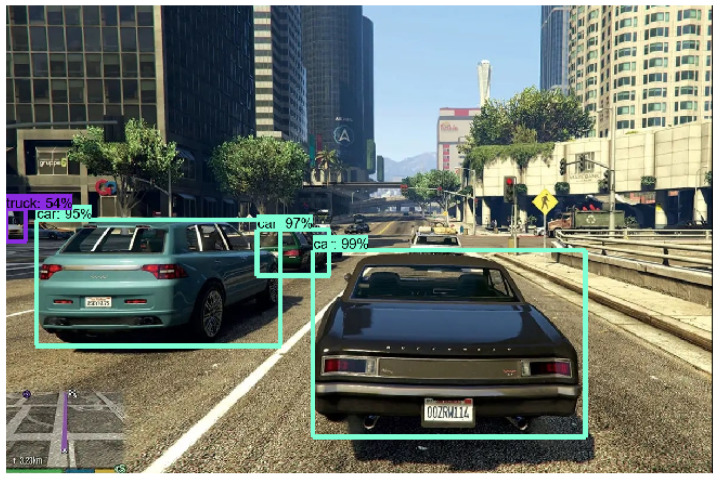
Image analysis through Faster R-CNN: car.

**Figure 9:**
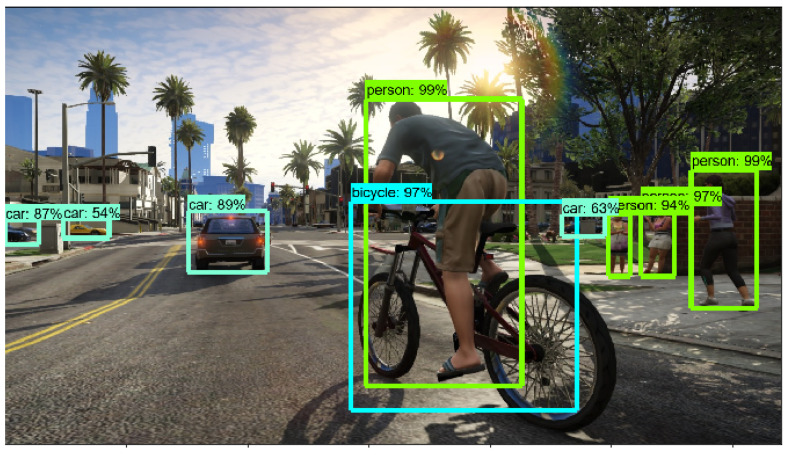
Image analysis through Faster R-CNN: object.

**Figure 10:**
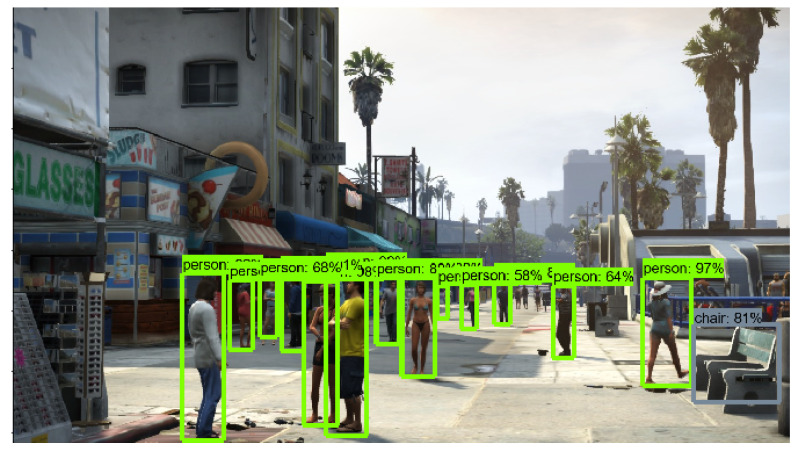
Image analysis through Faster R-CNN: pedestrian.

The implementation of the proposed model on a game environment results in precise object classification, which assists an agent in taking path-finding decisions avoiding obstacles. Figure 8, Figure 9 and Figure 10 show the output of object detection which is implemented on sample frames from the game environment. Here, identifying nearby cars, objects and pedestrians through Faster R-CNN depicts optimum accuracy and detecting these obstacles makes autonomous navigation more efficient.

**Figure 11:**
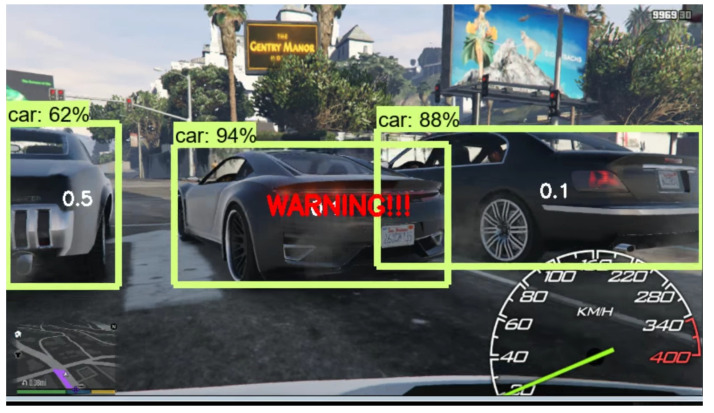
Braking situation in terms of distance calculation in GTA V environment.

Further, Table 2 calculates the braking distance measurement for the agent in the dry road condition. According to the calculated measures, an agent compares the braking distance with other surrounding cars. Parameter estimator algorithm (PEA) [31] has been incorporated for calculating the distance between our agent and other cars. Figure 11 portrays the braking situation in GTA V environment which is the visual representation of the braking distance calculation of the proposed algorithm. When the agent detects any car within the braking distance, it warns the agent. The agent then reduces the speed accordingly and finds a possible path to traverse.

**Figure 12:**
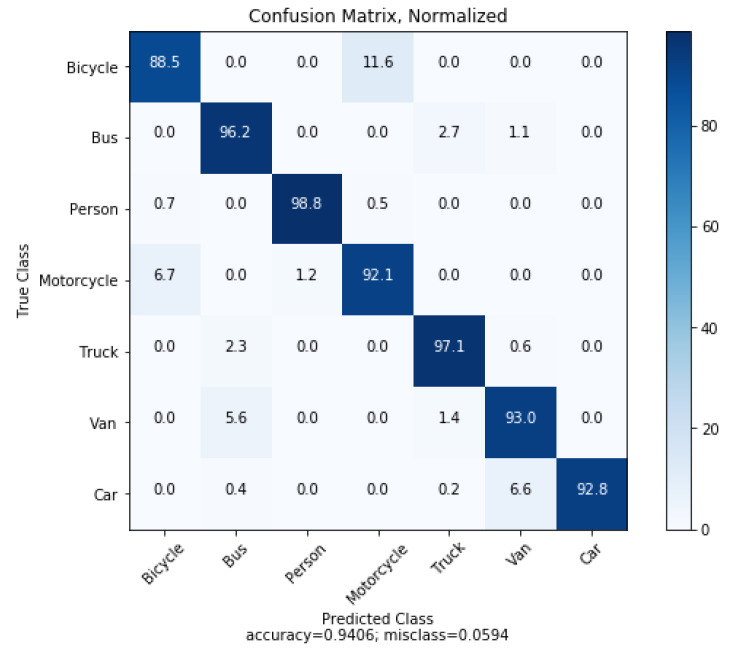
Normalized confusion matrix for the object classifications.

We run some tests on our model to evaluate the constructed object classifier. True negative and false positive values for each class defines the accuracy, and for evaluation, they require a normalized confusion matrix. We construct the matrix, and it is shown in Figure 12. Here, the rows represent the target values (what the model should have predicted—the ground-truth). The columns represent the predicted values (what the model predicted).

Furthermore, on a scale of 0.0 to 1.0, precision and recall for each class are computed gradually according to Bicycle, Bus, Person, Motorcycle, Truck, Van, Car (Seven distinct classes). Table 3 shows these values. Recall represents the capacity to find the relevant occurrences in a data-set, and precision depicts the fraction of the data points that the model says is relevant that is actually relevant. Overall results show that the image classification model is quite vital for detecting objects that will come in the way of our autonomous vehicle.

### Effect of Learning Rate Schedules

Among various learning rate schedule methods, the ReduceLROnPlateau callback method has been demonstrated which drops the learning rate by a factor after the monitored metric remains unchanged for a given number of epochs.

The impact of different ‘patience’ values can be explored here, where ‘patience’ value is the total number of epochs while waiting for a change before dropping the learning rate. Learning rate of 0.01 is used at first and dropped by an order of magnitude by setting the ‘factor’ argument to 0.1. It helps to observe the effect on the learning rate over the training epochs. This can be done by creating a new Keras Callback that is responsible for recording the learning rate at the end of each training epoch. The recorded learning rates can then be retrieved to plot a line graph to notice how the learning rate is affected by drops.

**Figure 13:**
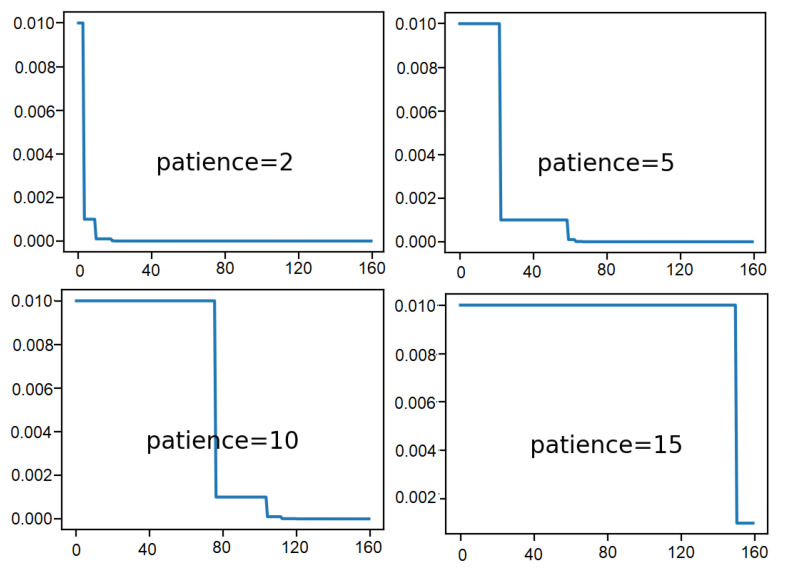
Result analysis of various patience values: learning rate over epochs.

Here, Figure 13 shows line plots of the learning rate over the training epochs for each of the evaluated patience values. A significant drop in the learning rate within 20 epochs can be observed when the patience value is the smallest. The learning rate only suffers one drop due to the largest patience value of 15.

From these plots, it can be expected that when the patience value is of 5 and 10, the model will result in better performance as a larger learning rate is allowed to be used for some time before the rate is dropped to refine the weights.

**Figure 14:**
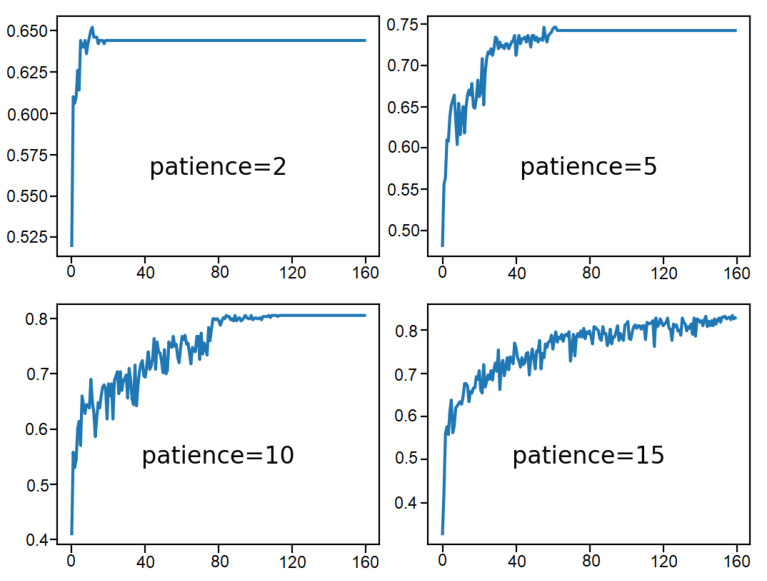
Result analysis of various patience values: training loss over epochs.

Secondly, Figure 14 indicates the loss on the training data-set for each of the patience values. The plot shows that the patience values of 2 and 5 initiate a rapid convergence of the model. It can possibly be up to a suboptimal loss value. In the event of patience values of 10 and 15, the loss value drops moderately until the learning rate also drops below a certain level where significant changes to loss value can be noticed. This happens halfway when the patience value is 10 and nearly at the end of the run in case of patience 15.

**Figure 15:**
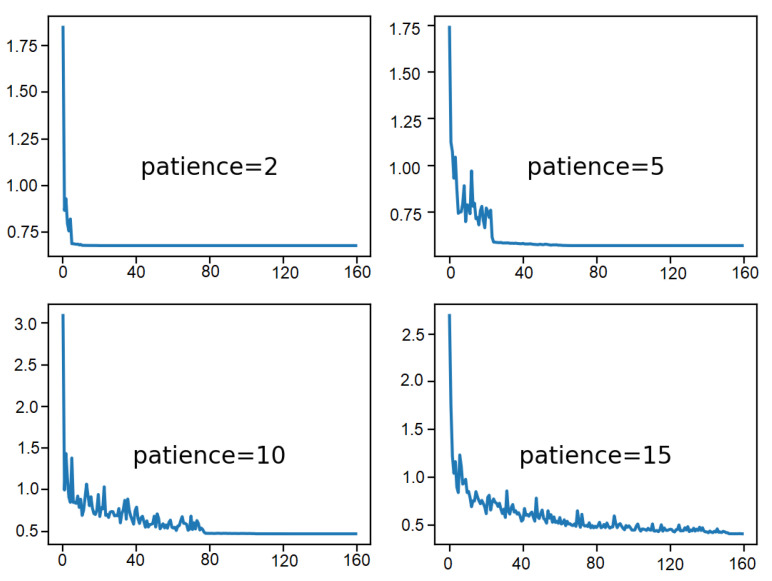
Result analysis of various patience values: training accuracy over epochs.

Lastly, Figure 15 shows the training set accuracy over training epochs for each patience value. It can be observed that, indeed, premature convergence of the model to a less-than-optimal model is resulted from the minor patience values of 2 and 5 epochs at around 65% and less than 75% accuracy, respectively. On the contrary, larger patience values result in better performing models, with the patience value of 10 that shows convergence just before 120 epochs. Patience value of 15 continues to show the outcome of a volatile accuracy given the close to unvaried learning rate.

Therefore, these plots represent how a learning rate that is decreased reasonably for the problem and the chosen model configuration can result in both a skillful and converged stable set of final weights as well as a preferable property in the final model at the end of the training run.

## 7. Discussion

This system works simultaneously with both Faster R-CNN and DDQN algorithms which makes the exploration process smoother. Implementing only a DDQN model limits the situation handling process of the agent. Therefore, the focus is kept more on the integration of both models to make the exploration process secure and effective without any interruption. Thus, it is important to normalize input data to remove unnecessary data. Further, the system’s accuracy and loss validation data are calculated simultaneously for checking the effectiveness of the algorithm. Comparison among the algorithm that we have used and other similar algorithms also depicts the system’s productivity.

**Figure 16:**
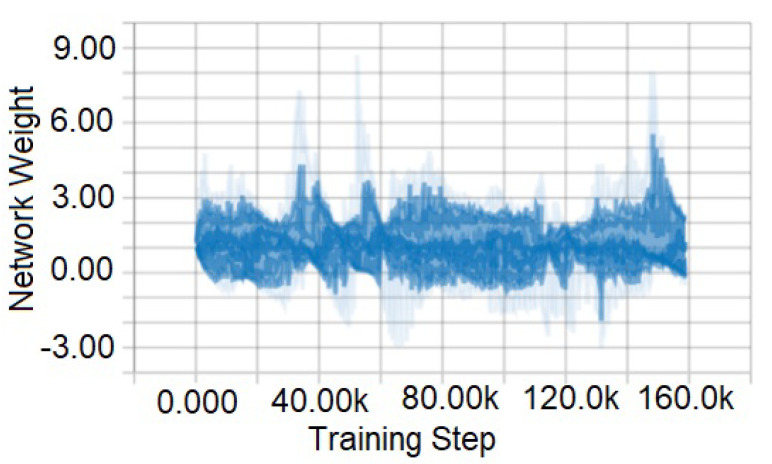
Training data before normalization.

**Figure 17:**
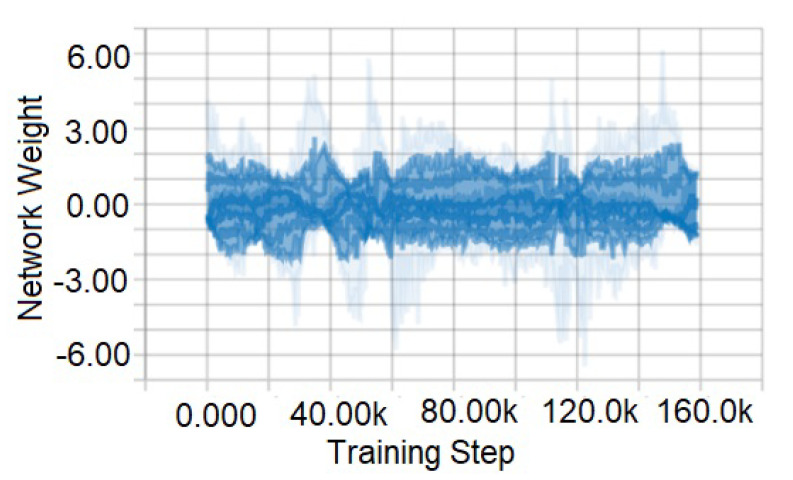
Training data after normalization.

Figure 16 and Figure 17 are the representation of training data collected from GTA V game environment. We test the system on the agent in possible scenarios that are close to a real-life environment (such as, night time, daytime, foggy, rainy, sunny, crowded etc.). Here, Figure 16 portrays a graph of raw training data. This data contains everything in the environment that has been captured. However, for obtaining accuracy and better performance, some data are entirely unnecessary. Therefore, raw data have been normalized, which is shown in Figure 17. This figure represents 160,000 normalized training data. By normalizing the data-set, more accurate results are obtained.

**Figure 18:**
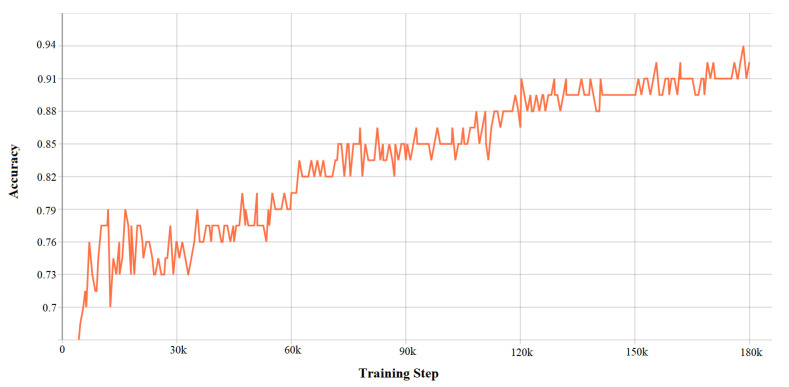
Characteristics of DDQN hyper-parameters: accuracy validation.

**Figure 19:**
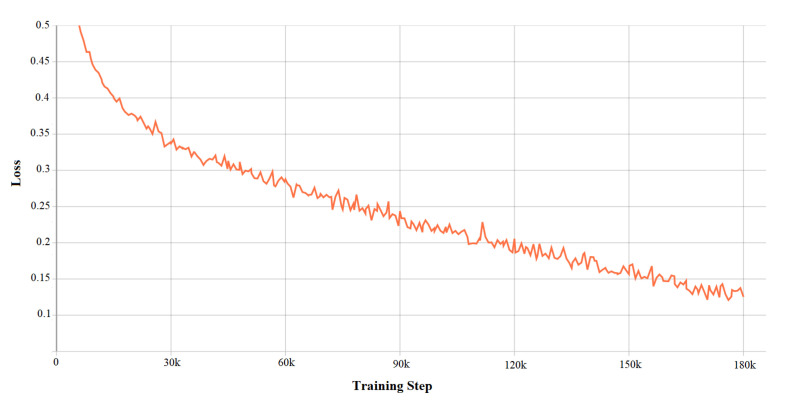
Characteristics of DDQN hyper-parameters: loss validation.

DDQN hyper-parameters determine the system’s performance. Tuning these parameters is thus, important to get better results from the developed algorithm. The following figures depict the characteristics of the DDQN hyper-parameter. Figure 18 defines the accuracy validation which comes from the accuracy validation function of Double Deep Q-Learning, and Figure 19 shows the total loss function from the values of the value loss function.

**Figure 20:**
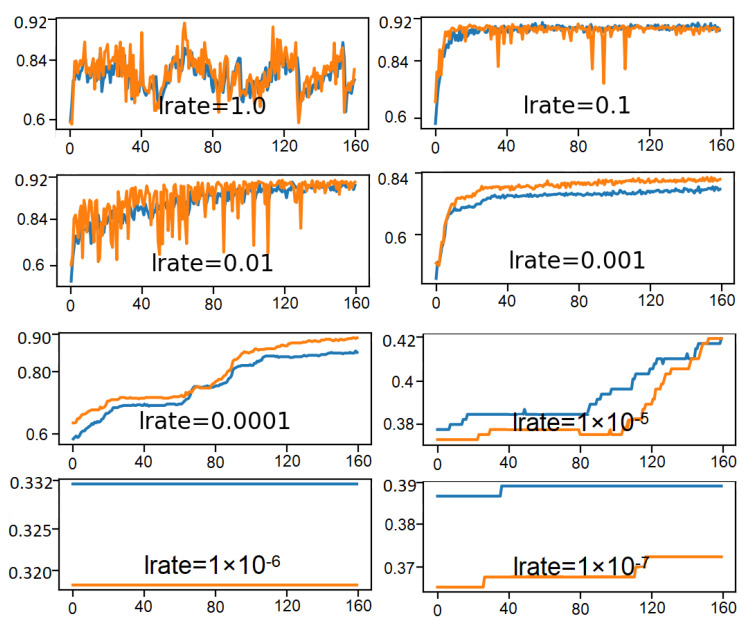
DDQN hyper-parameter tuning for learning rate.

Figure 20 illustrates eight line plots for eight different evaluated learning rates where epoch and accuracy values are represented respectively on the x-axis and y-axis. Classification accuracy of the training data-set is marked in blue, whereas accuracy of the test data-set is marked in orange.

The plots show oscillations in behavior for the too-large learning rates of 1.0 and the inability of the model to learn anything with too-small learning rates of 1 × 10${}^{-6}$ and 1 × 10${}^{-7}$. It can be seen that the model is able to learn well with the learning rates 1 × 10${}^{-1}$, 1 × 10${}^{-2}$ and 1 × 10${}^{-3}$, although successively slower as the learning rates were decreased. With the chosen model configuration, a moderate learning rate of 0.1 is suggested which results in satisfactory performance on the train and test sets.

The discount factor can dictate how far-sighted an agent can be. Values that are too insignificant will make an agent consider more about the reward that is present and values that are too big will make an agent pay the same attention to rewards after the time point. This may confuse the agent regarding which action leads to a high or low return.

**Figure 21:**
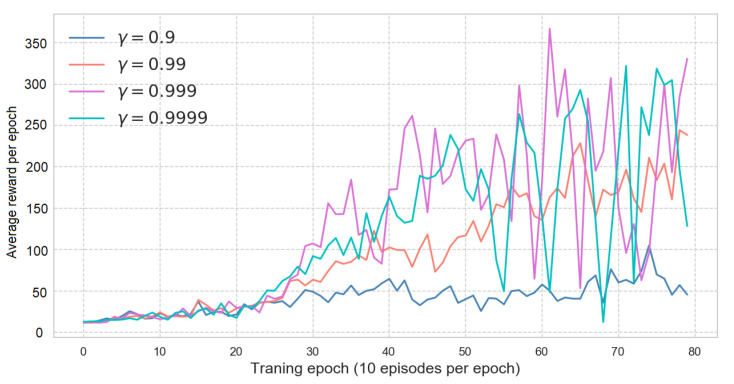
DDQN hyper-parameter tuning for discount factor, γ.

Figure 21 shows the average score for four different gamma values. It is evident that γ = 0.9 makes the agent short-sighted and there is no significant change during 80 epochs. When γ≥0.999, the average score fluctuates widely after the 50th epoch. Since γ = 0.99 has a gradually increasing trend, this is used as the final discount factor.

**Figure 22:**
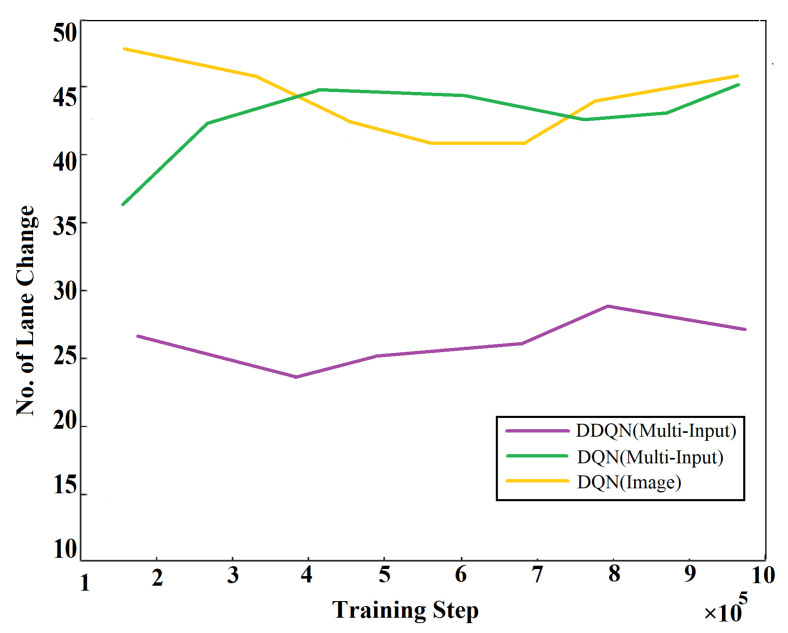
Lane changing comparison among different Q-Learning algorithms.

The effectiveness of the proposed algorithms is measured by comparing our implemented algorithm with two other similar Deep Reinforcement Learning algorithms. The compared algorithms are the Deep Q-Learning (Multi Input) and the Deep Q-Learning (Image), which only work on image processing. These algorithms are compared in terms of lane-changing decisions of an autonomous agent in GTA V game environment. Figure 22 shows the lane-changing results of these algorithms. The figure shows that the proposed algorithm proves to be more favorable than those algorithms. The number of lane changes refers to the compatibility of the algorithm. From the graph in Figure 22, it can be seen that the DDQN (Multi-Input) makes the lowest number of lane-changing decisions at the end of the training process. It indicates that the decision-making process is more precise in this algorithm. Hence, the proposed approach is best suited and reliable with Double Deep Q-Learning algorithm.

**Figure 23:**
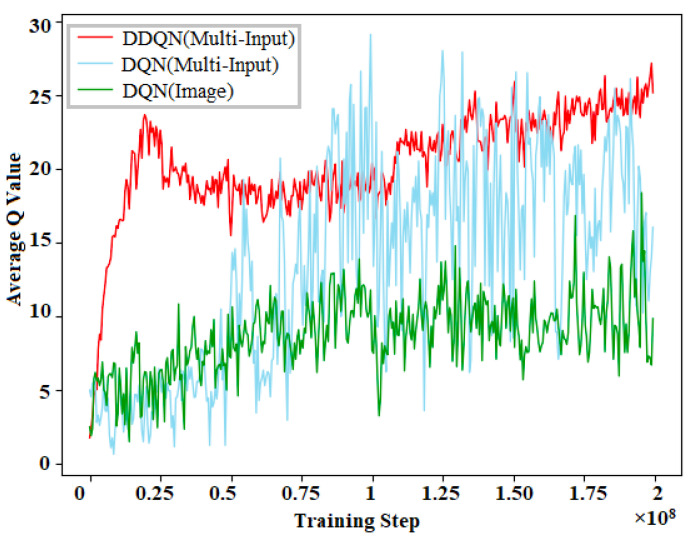
DDQN hyper-parameter attributes: average Q-value.

Reinforcement Learning gives reward based on Q-values which generate from Q function. That is why we compare different Q-Learning algorithms in terms of Q-values. Figure 23 shows the results of the Q-values of different Reinforcement Learning algorithms. The figure illustrates the comparison of the average Q-values of these algorithms, which is one of the characteristics of DDQN hyper-parameters. Q-Learning algorithms make a Q table based on Q-value for taking decisions. Q-Learning algorithms such as DDQN (Multi-Input), DQN (Multi-Input) and DQN (Image) have been considered for this comparison. This figure justifies that DDQN is more appropriate than other Q algorithms for autonomous vehicles. DQN (Image) is a Q-Learning algorithm which is only based on image processing showing the lowest average Q-value. DQN (Multi-Input) shows a better result than DQN (Image); however, its value is not consistent. That is why DQN algorithm for Multi-Input can give inaccurate results at the time of decision-making. On the contrary, DDQN algorithm for Multi-Input represents the maximum average Q-value with a consistency which makes the algorithm more efficient than the rest.

**Figure 24:**
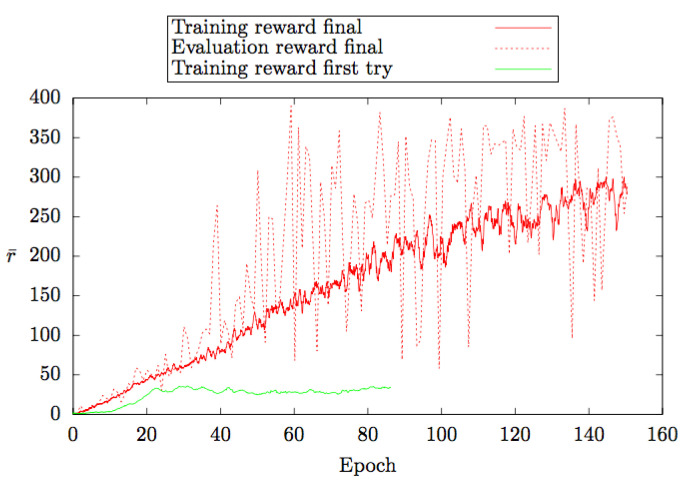
DDQN hyper-parameter attributes: average reward value.

The graph in Figure 24 is generated from the average reward values ($\bar{r}$) of our DDQN hyper-parameter. In this figure, three values (training reward final, evaluation reward final and training reward with first try) have been considered which have been incorporated for the uninterrupted traversing of the agent. The green line shows the training reward values in the first try, which is very low. Better reward values have been obtained in the training process through trial and error, which is shown by the red dotted line. Furthermore, by normalizing the evaluation reward values, the deep red line has been found, which is the final reward values in the training process. The DDQN algorithm works based on the final reward values in the game environment. The final reward value increases at a consistent rate which shows the precision of the proposed algorithm.

## 8. Conclusions

This work develops a model that integrates the Double Deep Q-Learning (DDQN) algorithm with Faster R-CNN in autonomous vehicles for making decisions to navigate avoiding obstacles on its way. DDQN algorithm has a reward system policy for which a vehicle can take maneuvering decisions in a stochastic environment. Thus, DDQN algorithm ensures more effectiveness over other non-distributional algorithms. Additionally, where most of the existing models for the autonomous vehicle are entirely constructed on the frame of neural networks, our proposed model integrates Faster R-CNN with DDQN to detect any object that appears in front of the autonomous vehicle. Our proposed model, thus, can stimulate accuracy in safe and smooth decision-making even in unexpected situations. Unlike the existing systems, the proposed model is not only enclosed in a mapped environment. Accordingly, our model can avoid the limitations exhibited by the existing systems on exploration scope and Precision. However, the model has potential limitations that include less number of integrated sensors as the system only uses vision sensors to take data from the environment. The system has scope to perform better in stochastic environments if integrated with more sensors. Further, the system faces challenges in decision-making when it has to consider the vehicles which are in parallel sides. Testing the algorithms on a real vehicle requires a large amount of environmental data of a dynamic environment which is expensive and time consuming. A real autonomous vehicle might face challenges to perform globally considering different infrastructural differences of different countries.

Although the proposed model attains preferable results, the key recommendation to work on this system in the future is to integrate more sensors like Lidar and Sonar to make it more efficient regarding decision-making and accurate in path-finding by detecting obstacles. In the future, we hope to overcome the limitation of resources to implement the system practically on a real vehicle. In addition, we expect to increase the accuracy in decision-making during the time of any randomness and to take preferable action complying with reality. Further, the object classifier also has great scopes for improving its performance by lowering the average number of misclassification and by increasing the average number of accuracy levels. By configuring the confusion matrix; it will simply indicate the percentage of accuracy that has been acquired through these processes. Therefore, in the future, we hope to increase the iteration score while training object classes to overcome the misclassification problem.

## Figures and Tables

**Table 1:** Autonomous propulsive approach network: hyper-parameters.

Input	Layer Nature	Actuation	Hyper-Parameters of Strategy Network
			(5 × 5) Patch size
			4 Strides
			24 filters
			(5 × 5) Patch size
			4 Strides
			36 filters
			(5 × 5) Patch size
Vision Sensor Data	Convolution 2D	ReLU	4 Strides
			48 filters
			(3 × 3) Patch size
			1 Strides
			64 filters
			(3 × 3) Patch size
			1 Strides
			64 filters
Integrated Data	Fully Connected Layer	ReLU	512 Units

**Table 2:** Braking distance calculation of dry road.

Speed	Reaction Distance	Braking Distance	Total Stopping Distance
80 km/h	33 m	36 m	69 m
70 km/h	29 m	27 m	56 m
60 km/h	25 m	20 m	45 m
50 km/h	21 m	14 m	35 m
40 km/h	17 m	9 m	26 m

**Table 3:** Gradual precision and recall values of various classes.

Class	Precision	Recall
Bicycle	0.89	0.94
Bus	0.97	0.93
Person	1.0	0.99
Motorcycle	0.93	0.89
Truck	0.98	0.97
Van	0.94	0.93
Car	0.94	1.0

## Data Availability

Data sharing not applicable.

## Abbreviations

The following abbreviations are used in this manuscript:

CNN	Convolutional Neural Network
CUDA	Compute Unified Device Architecture (Nvidia)
DDQN	Double Deep Q-Network
DQN	Deep Q-Network
GTA	Grand Theft Auto
MDP	Markov Decision Process
PEA	Parameter estimator algorithm
R–CNN	Region based Convolutional Neural Network
ReLU	Rectified Linear Unit
ROI	Region of Interest
RPN	Region Proposal Network
CSV	Comma-Separated Values
